# Grating-Based Phase-Contrast Imaging of Tumor Angiogenesis in Lung Metastases

**DOI:** 10.1371/journal.pone.0121438

**Published:** 2015-03-26

**Authors:** Huimin Lin, Binquan Kou, Xiangting Li, Yujie Wang, Bei Ding, Chen Shi, Huanhuan Liu, Rongbiao Tang, Jianqi Sun, Fuhua Yan, Huan Zhang

**Affiliations:** 1 Department of Radiology, Ruijin Hospital, School of Medicine, Shanghai Jiao Tong University, Shanghai 200025, People’s Republic of China; 2 Department of Physics, Shanghai Jiao Tong University, Shanghai, 200240, People's Republic of China; 3 School of Biomedical Engineering, Shanghai Jiaotong University, Shanghai 200240, China; H. Lee Moffitt Cancer Center & Research Institute, UNITED STATES

## Abstract

**Purpose:**

To assess the feasibility of the grating-based phase-contrast imaging (GPI) technique for studying tumor angiogenesis in nude BALB/c mice, without contrast agents.

**Methods:**

We established lung metastatic models of human gastric cancer by injecting the moderately differentiated SGC-7901 gastric cancer cell line into the tail vein of nude mice. Samples were embedded in a 10% formalin suspension and dried before imaging. Grating-based X-ray phase-contrast images were obtained at the BL13W beamline of the Shanghai Synchrotron Radiation Facility (SSRF) and compared with histological sections.

**Results:**

Without contrast agents, grating-based X-ray phase-contrast imaging still differentiated angiogenesis within metastatic tumors with high spatial resolution. Vessels, down to tens of microns, showed gray values that were distinctive from those of the surrounding tumors, which made them easily identifiable. The vessels depicted in the imaging study were similar to those identified on histopathology, both in size and shape.

**Conclusions:**

Our preliminary study demonstrates that grating-based X-ray phase-contrast imaging has the potential to depict angiogenesis in lung metastases.

## Introduction

Angiogenesis is traditionally known as the growth of new capillary blood vessels from preexisting ones. Recently, it is also suggested that these vessels can originate from cells recruited from the bone marrow or can differentiate from tumor stem cells [1]. Because new blood vessels carry nutrients and oxygen into tumors and transport catabolites and carbon dioxide away from them, angiogenesis plays a critical role in the growth of cancer [2,3], from the initial growth to a clinical detectable size, to the development of a metastatic or lethal phenotype, until eventually killing its host [4,5,6,7,8]. Because angiogenesis is essential for tumor biology, the redundancy and diversity of blood vessel remodeling might be responsible for the poor efficacy of or acquired resistance against anti-angiogenesis therapies [1]. Treatment efforts have been made to disturb this process [9,10]. Consequently, these therapies have inspired many research activities in the assessment of tumor vascularity to monitor therapeutic effects, *e*.*g*., histological assessment of microvessel density (MVD) and angiogenesis imaging.

According to statistics, metastases cause 92% of cancer deaths [11]. Angiogenesis is also important for metastasis. During the metastatic process, tumor cells must migrate into the blood vessels from the primary tumor, survive the circulation, settle at the microvasculature of the target organ while evading its vasculature, and finally induce angiogenesis [6]. Metastases to the lung, which is one of the most frequent metastasis sites, account for 25–30% of all patients with metastasis at autopsy [12]. Therefore, imaging of angiogenesis in lung metastases has been considered crucial in the diagnostic imaging of metastatic lung tumors.

Compared to MVD, noninvasive and repeatable angiogenesis imaging methods are gaining more popularity as tools to monitor tumor growth, vessel recruitment, and metastasis [10,13]. Angiogenesis imaging can be divided into two categories including functional and anatomical imaging. Relying on the binding of labeled molecules and highly expressed markers on the endothelium of the tumor vasculature, receptor-targeted imaging can characterize the status of the endothelium cells. However, the synthesis of effective compounds remains a big challenge [14].

Conventional medical imaging employs strong X-ray-absorbing contrast medium to enhance the contrast in the imaging of weakly absorbing materials, such as blood vessels. With the aid of contrast agents, computed tomography (CT) revealed the internal irregular vessels of liver metastasis and various other metastasis enhanced at different phases depending on their various primary tumors [15]. Preclinical micro-CT, with a spatial resolution of 50–100 μm *in vivo* and up to 10 μm *ex vivo*, overcomes the limitation of CT technologies for depicting small blood vessels [16]. HY *et al*. depicted the morphological characteristics of microvessels, the microcirculation of the metastatic tumor, and the surrounding hepatic sinusoids by micro-CT. They found that when metastases grow to a size about 500–1,500 μm in diameter, the diameters of the tumor vessels also increase to approximately 22.4 μm, and the tumor vessels are spatially distributed in a heterogeneous fashion and appear heterogeneous from normal vessels [17]. However, the contrast agents ordinarily employed during micro-CT, containing iodine-based non-ionic extracellular water-soluble compounds and are rapidly cleared from the blood stream within seconds after injection. This becomes a severe problem for imaging in rodents because the clearing speed is at least 10 times faster than in humans. This leads to insufficient contrast agent to cover a full scan [18]. In addition, the potential contrast agent-related allergic reaction and renal impairment due to contrast agent contraindication should also be taken into account [10]. Micromagnetic Resonance Imaging (Micro-MRI) claims a higher spatial resolution of 100 μm with improved contrast sensitivity [19]. However, similar to micro-CT, micro-MRI requires gadolinium (Gd)-based or iron-oxide-based magnetic contrast agents to achieve contrast enhancement in visualizing blood vessels. Furthermore, MRI is more sensitive to susceptibility artifacts as compared to CT [10]. The most frequent adverse reactions associated with the contrast agents include transient headache, nausea, and emesis. Besides, allergic reactions have been reported as well [20,21,22].

Unlike conventional medical imaging, phase-contrast imaging (PCI) employs the phase shift generated by X-rays passing through the object as the phase contrast mechanism [23,24]. As the interaction cross section of the X-ray phase shift is about a thousand times larger than that of the absorption for soft tissues, PCI can be very effective in the imaging of soft tissues [25]. A study reported that PCI depicts blood vessels down to the micron level, without the aid of staining [26]. Currently, PCI is classified into interferometric imaging [26,27], in-line imaging [28,29], diffraction-enhanced imaging (DEI), and grating-based phase-contrast imaging (GPI) [30,31,32]. Most of the PCI methods require the coherence of synchrotron X-rays, have rather limited field-of-views, and cannot be applied to lab X-ray sources. Therefore, until now, the application of PCI in medical study is rather limited [32].

It has been demonstrated that GPI, using gratings as the optical elements, is compatible with a conventional, low-brilliance X-ray source, which is considered a breakthrough in phase-contrast imaging. In other words, its requirements for temporal and spatial coherence are moderate. Additionally, GPI is an ideal method for soft tissue imaging owing to its sensitivity to shallow phase gradients [32]. Attributing to these characteristics, GPI is currently a hot topic in medical imaging research and rapid advances are being achieved. Early technique evaluation studies have mainly focused on the normal tissues [33,34,35]. With the rapid advancement of the field, more realistic medical applications based on GPI have been carried out in recent years, including studies on various tumors, such as invasive ductal carcinoma, ductal carcinoma in situ, and brain tumors [36,37]. In terms of vessel imaging with GPI, prior studies mainly concentrated on normal liver tissues [38,39,40,41], and there has been a lack of relevant well-established studies on pathological angiogenesis.

In the current study, we employed GPI with a synchrotron source to demonstrate the feasibility of phase-contrast imaging technique for tumor angiogenesis. Reports show that synchrotron, with the best beam coherence in terms of both spatial coherence and spectral width, is ideal for high-resolution tomography with high contrast in small samples, which is beneficial for our purpose of assessing the feasibility of using GPI to observe tumor angiogenesis in lung metastases without the use of contrast agents.

## Materials and Methods

### Ethics Statement

This study was conducted according to the standards established by the Guidelines for the Care and Use of Laboratory Animals of Shanghai Jiao Tong University, and it was approved by the Laboratory Animal Ethics Committee of Rui-Jin Hospital (Permit Number: 112). Mice were anesthetized using an intraperitoneal injection of sodium pentobarbital anesthesia to minimize suffering.

### 3.1 Cell line, animal model and sample preparation

The human moderately differentiated SGC-7901 gastric cancer cell line [42,43] was obtained from the Chinese Academy of Sciences in Shanghai. Cells were propagated in RPMI-1640 media supplemented with 10% fetal bovine serum, 100 units/ml penicillin, and 100 μg/ml streptomycin, at 37°C under 5% CO_2_. Exponentially growing cells were harvested with trypsin/EDTA (Invitrogen) at a concentration of 1x10^6^/ml. Six-week-old male athymic BALB/c (nu/nu) mice were purchased from the Animal Center, CAS, Shanghai, China, and kept in standard animal cages under specific pathogen-free conditions in the animal facility. Then, 0.1 ml suspensions of SGC-7901 cells were injected into the mice tail veins to establish the lung metastasis model. After 4 weeks, the mice were euthanized with an overdose of 10% pentobarbital sodium. The lungs were harvested and embedded in 4% formalin for 72 hrs at 4°C to allow ample fixation time [44]. Subsequently, the lung samples were brought to SSRF for CT scanning. In order to fit the FOV, 1.2 × 4 × 3.5 mm^3^ and 5.1 × 6.9 × 5.7 mm^3^ pieces, consisting of healthy lung tissue and metastasis, were cut from the corresponding two samples. Afterwards, the samples were rubbed dried completely and placed in plastic containers for further processing.

### 3.2 Grating based phase-contrast imaging with SR X-ray

#### 3.2.1 Image acquisition

**Fig 1:**
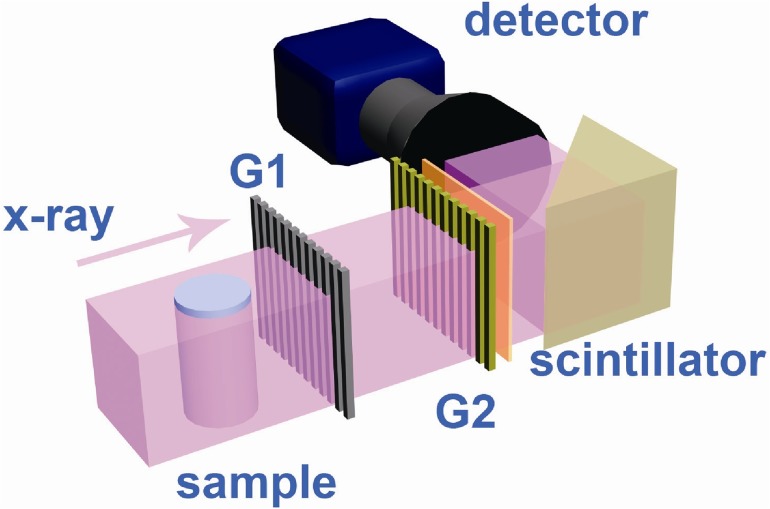
Schematic of the grating interferometer set-up at the BL13W beamline of SSRF.

The synchrotron experiments were performed at the X-ray imaging and biomedical application beamline (BL13W1) at the Shanghai Synchrotron Radiation Facility (SSRF). Emitted from a wiggler source, the X-ray source is approximately 400(H) μm×24 (V) μm and is located 30 m away from the sample. The typical GPI-CT set-up employed in our study is shown in Fig. 1. The grating interferometer setup comprised one phase grating (G1) and one absorption grating (G2). G1 was composed of nickel strips on the silicon substrate with a 2.4 μm period and introduced a π/2 phase shift. G2 was composed of gold strips on the silicon substrate and shared the same period with G1. The phase grating G1 acts as a beam splitter, dividing the incident beam essentially into two diffraction orders, which form a periodic interference pattern in the plane of the analyzer grating G2 downstream. Absorption grating G2 functions as a transmission mask for the detector, transforming phase modulation induced by G1 into an intensity modulation[45]. Both gratings were manufactured by Microworks, Inc. (Karlsruhe, Germany) and used at the first-order Talbot distance of 4.64 cm at the X-ray energy of 20 keV [46]. For the first sample, the imaging system was placed immediately after the absorption grating, which was composed of a PCO.Edge scientific CMOS camera (PCO AG, Kehlheim, Germany) coupled with a 3× objective and a 200-μm-thick YAG:Ce scintillator. The effective pixel size was 2.2 μm and the field of view (FOV) was 5.6 mm×3.5 mm due to the vertical limitation of the beam size and horizontal limitation of the number of pixels of the camera. The exposure time for each image was 300 ms. The second sample was scanned with an X-ray CCD camera (Photonic Science, UK) with an effective pixel size of 9 μm. The FOV was 13 mm×3.5 mm, and the exposure time was 5 ms.

We collected 720 projection angles for a 180° rotation and 10 reference projections without a sample every 90 projections in both CT scans. In each projection, the phase grating was scanned in six steps over one grating period. The entire CT imaging process required 40 min for the first sample and 2 hours for the second one.

#### 3.2.2 Image reconstruction

In the current study, we utilized the phase-stepping method to separate the phase information from other contributions. G1 was scanned along the transverse direction, perpendicular to both the X-ray beam and the grating line directions, over one period of the gratings. A corresponding image was acquired at every point of the scan [45].

With the phase-stepping method, the intensity function for each detector pixel (p_x_, p_y_) can be written as follows:

$$I(p_{x},p_{y},x_{g})=\sum_{i}a_{i}(p_{x},p_{y})\text{cos}(\frac{2\pi }{p}x_{g}+\varphi_{i}(p_{x},p_{y}))\approx a_{0}(p_{x},p_{y})+a_{1}(p_{x},p_{y})\text{cos}(\frac{2\pi }{p}x_{g}+\varphi_{1}(p_{x},p_{y}))$$

where x_g_ incorporates the grating position, a_i_ represents the amplitude coefficients, and p is the grating period. The phase coefficient *φ*_1_ is directly proportional to the gradient of the integrated real part of the refractive index δ along the X-ray direction as: $\varphi_{1}=\frac{2\pi d}{p}\frac{\partial }{\partial x'}\int \delta (x',y')dy'$ where d is the Talbot distance. The Hilbert-filter-based filtered back projection (FBP) algorithm was used for data reconstruction [47]. A combined wavelet-Fourier filter was employed to reduce the ring artifacts [48]. The reconstructed images were mapped on a linear gray value scale for optimal demonstration of vessels and tumors.

#### 3.2.3 Data analysis

3D tomography images were manually reoriented to match the histological section, and the vessels were identified by two experienced radiologists. A CNR (Contrast-to-noise ratio) analysis was performed to quantify the contrast significance between the tumor and the vessels. Three homogeneous ROIs (region of interest) were selected in each image, including 1) tumor (red square), 2) vessel (yellow square) and 3) the background region (blue square). The CNRs were calculated as follows:

$$CNR=\frac{\left|M_{tumor}-M_{vessel}\right|}{\sigma_{background}}\;,$$ (1)

where *M*_*tumor*_ and *M*_*vessel*_ are the mean gray value of the tumor and vessel regions, respectively, and *σ*_*background*_ is the standard deviation of the gray value in the background region. The uncertainty of the CNR was determined using the standard error propagation method[49].

## Histology

After the locations and orientations of the suspected vessels were identified in the reconstructed GPI-CT tomogram, the corresponding parallel histological sections were selected after taking into account the general shapes of the samples. The samples were embedded en bloc in paraffin, and then performed with a standard hematoxylin and eosin (H&E) staining. The slice thickness was approximately 4 μm (Leica RM2235, Germany). The cancer cells from the lung tissues were confirmed by two pathologists. The coregistration of the histology section with the GPI tomogram was carried out based on peculiar image features, including the relative distances between the bronchi around, and gross morphological features, such as the size and shape of the tumors and the peripheral bronchi. The validity of the coregistration was confirmed based on the consistency of the blood vessel diameters measured independently from the H&E slices and the gray value chart in the GPI tomogram [50].

## Results

**Fig 2:**
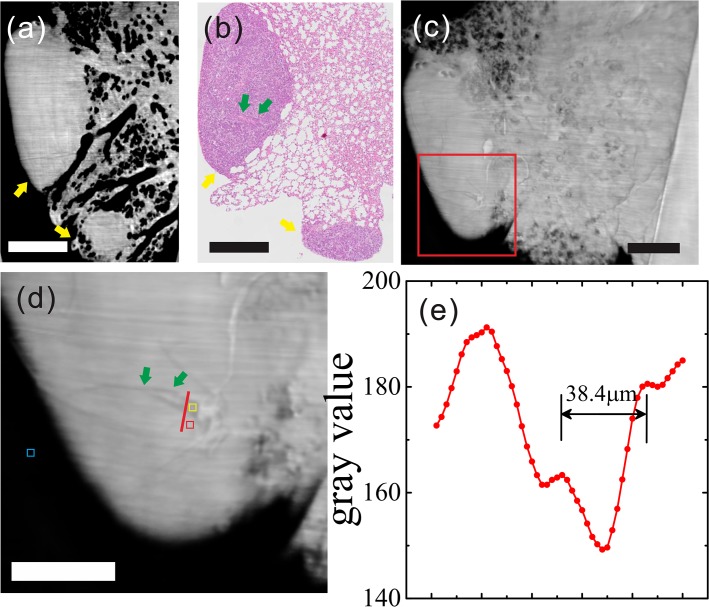
Reconstructed tomogram of lung metastasis sample (sample one) from HE pathology, GPI-CT, MIP and gray value graphs. (a) Reconstructed tomogram in GPI-CT, yellow arrows: tumor lesion. (b) Histological section: yellow arrows: two lung metastatic tumors; green arrows: microvascular structures in the tumor. (c) MIP. (d) Enlarged view of the red box in (c), which reveals the presence of two blood vessels with blood cells inside. Three ROIs were selected, including 1) tumor (red square), 2) vessel (yellow square) and 3) the background region (blue square). The gray values along the red dashed lines across the blood vessels in (d) are plotted in (e). (a-c scale bar: 600 μm; d scale bar: 300 μm).

**Fig 3:**
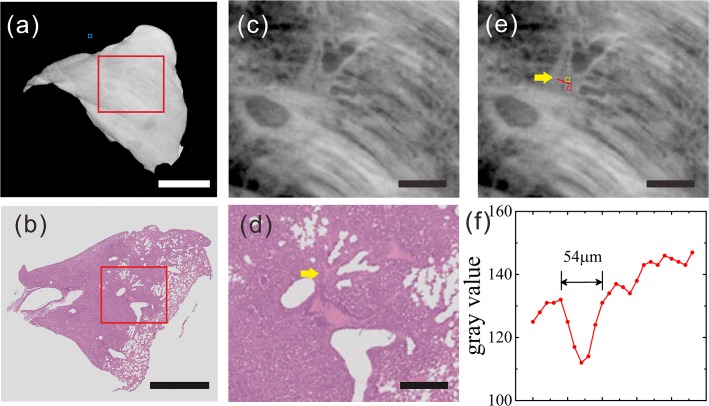
Reconstructed tomogram of lung metastasis sample (sample two) from HE pathology, GPI-CT and gray value graphs. (a) Reconstructed tomogram in GPI-CT. (c, e) Enlarged view of the red box in (a), which indicates the presence of branch-like blood vessels with blood cells inside within the tumor. Three ROIs were selected, including 1) tumor (red square), 2) vessel (yellow square) and 3) the background region (blue square). (b) Histological section. (d) Enlarged view of the red box in (b); yellow arrow: blood vessel. The gray values along the red dashed lines across the blood vessel in (e) are plotted in (f). (Scale bar in (a) and (b): 2 mm; Scale bar in (c), (d) and (e): 500 μm).

Because only a few vessels were found in the samples, as shown in the histological sections, we have exploited two sections that could be matched to the CT images [Figs. 2 and 3].

### 5.1 Results of Sample one

The tumors could be visualized with a rather high contrast in the sagittal plane of the reconstructed tomogram of the sample [Fig. 2A]. The structure of lung tissue besides the two tumor lesions were also clearly revealed owing to the high imaging resolution (effective pixel size of 2.2 μm). Lots of bronchioles and alveoli were visible in the reconstructed images. In the maximum intensity projection (MIP) [Fig. 2C], with a slice thickness of approximately 300 μm, two blood vessels were identified in one of the tumor lesions, with a better visibility in the enlarged view [Fig. 2D]. Without contrast agent, there was a significant difference in the gray values between the blood vessels and the surrounding tumor tissue [Fig. 2E]. The measured diameter 38.4 μm was similar to the 45 μm obtained with low-magnification (×10) optical microscopy.

### 5.2 Results of Sample two

In terms of the overall shape, the cross section from the GPI-CT matched the histological section [Fig. 3A-3B]. Similarly, without contrast agent, the blood vessels within the tumor identified by GPI, as shown in the enlarged view, was in agreement with the results of the histological sections, both in shape and in size [Fig. 3C-3D]. We employed the gray value as the quantitative measure for this sample as well. According to the gray value chart, the measured size of the vessel was 54 μm, which was close to the 45 μm value from the histological section [Fig. 3F]. Again, cross sections of the bronchioles and alveoli were clearly visible despite the relatively lower spatial resolution as compared with sample one. Relevant imaging conditions of the two samples including CNR and doses are listed in Table 1.

**Table 1:** Relevant imaging conditions of two samples.

	ROI Area (μm2)	CNR	Dose (Single projection)	Accumulated Dose
Sample 1	35*35	5.03±0.35	11.75mGy	50.76 Gy
Sample 2	54*54	3.22±0.71	0.68 mGy	2.94 Gy

## Discussion

In the current study, without using any contrast agent, blood vessels within lung metastases were demonstrated using GPI, which were shown in clear contrast from the surrounding tumors. The results were consistent with histological sections. Furthermore, vessels without additional management are exploited, close to that in physiological status. This study indicates the feasibility of GPI in the study of lung angiogenesis.

Traditionally, due to the small differences in the X-ray absorption coefficients between the vessel wall and the surrounding tissue, vessels have always been depicted using contrast agents containing a heavy element in conventional absorption imaging [40]. With the development of various phase-contrast imaging methods, experiments have been carried out to assess the feasibility of PCI in angiography, mainly using interferometer and in-line PCI. Momose *et al*. first depicted blood vessels of the excised rodent liver with physiological saline using phase-contrast X-ray computed tomography with an X-ray interferometer [41]. In a subsequent study, Takeda *et al*. showed vessels filled with physiological saline, down to approximately 30 μm in excised rat liver using the interferometer. The corresponding absorption-contrast X-ray images showed no vessels at all. Even with iodine-loaded acrylic microspheres as the contrast agent, only large portal veins 100 μm in diameter could be depicted [40]. Later, physiological saline-infused hepatic vessels of about 60 μm in diameter were demonstrated *in vivo* using the interferometer [38]. Similar to interferometric PCI, in-line PCI also revealed fine excised hepatic vessels down to 30 μm with physiological saline [39]. Besides physiological saline, barium sulfate with a persistent contrast-enhancement effect has also been employed together with in-line PCI, which showed great potential in the study of murine hepatocellular carcinoma microangiography [51,52]. All these PCI studies show great potential for angiogenesis imaging, with a sensitivity that is much higher than that of absorption imaging. However, despite its excellent performance, interferometric imaging requires the use of sophisticated X-ray optics, including perfect crystal alignment and mechanical stability. For in-line phase-contrast imaging, a very high degree of spatial coherence is indispensable. Because most of the prior studies were based on synchrotron, the corresponding fields of view are rather small [53]. Additionally, all of these previous studies depicted the vessels either with the aid of physiological saline or barium sulfate. The consequences are that no further histological analysis can be conducted after the injection of barium sulfate [51,52], and the perfusion of physiological saline with vessels cannot be utilized in clinical imaging. As a matter of fact, compared to these previous studies, the blood-containing vessels found in our study, without any contrast agent injection, are closer to that in physiological status.

It is worth mentioning that one type of contrast agent, Gold nanoparticles (AuNPs), could be a viable alternative to angiogenesis imaging. Gold nanoparticles have enhanced chemical stability, long circulation times, and obvious advantages over small-molecule iodinated contrast agents in terms of its low renal toxicity [54]. Chien *et al*. demonstrated the usage of AuNPs for studying tumor angiogenesis in a synchrotron imaging experiment by imaging vessels of 40 μm. However, although AuNPs are largely nontoxic and biocompatible, the high cost of gold is one potential limitation [54,55].

Recently, Xi *et al*. demonstrated the carotid artery and carotid vein in a formalin-fixed mouse in situ [46] using GPI. In the current study, we studied a more realistic medical imaging application of the angiogenesis process in lung metastasis. The study demonstrated that GPI can depict vessels inside the tumor without the aid of contrast medium, which can benefit the diagnostic imaging of lung metastasis in the future.

In the current study, we used two different imaging setups. The first setup is a high-spatial resolution setup that can clearly depict blood vessels measuring tens-of-microns in diameter. However, this high spatial resolution required a high dose, as shown in Table 1. To achieve a compromise between resolution and dose, a direct detection X-ray detector was employed in the second setup, which had much higher X-ray detective efficiency but slightly lower spatial resolution. As a result, the exposure time was dramatically decreased, and the accumulated absorption dose of Sample 2 was less than the average lethal dose for *in vivo* imaging for a living rat, while the vessels inside the tumor were still visible.

It is important to note that some improvements can be applied to further reduce the radiation dose in future research: 1) improvements in the detection efficiency of the image acquisition system to decrease the exposure time, which includes using a CCD detector with a higher quantum efficiency and an objective with a larger numerical aperture; 2) advanced acquisition schemes such as reverse projection methods and sliding window interlaced methods to reduce the stepping numbers [32,56]; and 3) a compressed sensing (CS) inspired iterative reconstruction algorithm can be used to drastically reduce the projection sampling number [57]. Nonetheless, in the current study, we were engaged in the feasibility of GPI for the study of lung metastasis angiogenesis. This study represents the first step towards future *in vivo* studies.

It should be noted that our image quality can be further improved through optimizing the reconstruction algorithm and the hardware. Because of the limitation of the source coherence, we used the first-order Talbot distance in the grating interferometer setup. The visibilities of the samples were rather weak. The source grating will be introduced in future research, and high-order Talbot distances will be available in the GPI setup, which can improve the system’s density resolution and enhance the visibility of the samples.

In addition, the thickness of the histological section is about 4 μm, which is much less than that of the MIP (300 μm) in the sample one. In other words, MIP represents a composite of nearly 75 histology sections. That means that the co-registration is less precise in Fig. 2. Again, it explains why two vessels were found in MIP and only one vessel was observed in the histological section, to some extent. This is a limitation of the study and requires further improvement.

For our future study, more *in vivo* samples need to be investigated to determine the potential of GPI-CT for studying tumor angiogenesis. In addition, we will be engaged in the quantitative analysis of the refraction indices to discriminate bronchioles from vessels inside tumors, which appear quite similar in GPI. Additionally, we will exploit blood vessel special markers, such as CD31, to confirm the target vessels, rather than depending on H&E staining, which only depicts vessels based on morphology. By comparing with MVD, quantitative analysis of vessels could be implemented in a further step.

## Conclusions

In conclusion, our preliminary study demonstrates that grating-based X-ray phase-contrast imaging has the potential to depict the angiogenesis of lung metastasis.
